# Zhi-zi-chi decoction exerts hypnotic effect through gut-brain axis modulation in insomnia mice

**DOI:** 10.3389/fmicb.2025.1645190

**Published:** 2025-08-07

**Authors:** Qianqian Wang, Boyi Zhang, Zixuan Guo, Jiawei Huang, Yuanyuan Niu, Junhong Huang, Yu Huang, Dandan Guo, Baiyan Wang, Shuying Feng

**Affiliations:** ^1^Medical College, Henan University of Chinese Medicine, Zhengzhou, China; ^2^Henan Engineering Research Center for Chinese Medicine Foods for Special Medical Purpose, Zhengzhou, China

**Keywords:** Zhi-zi-chi decoction, insomnia, gut-brain axis, medicinal and edible homologous food, gut microbiota

## Abstract

**Background:**

Insomnia, a frequently encountered sleep disorder, boasts a high prevalence rate on a global scale. Currently, Western medications are widely used for its treatment; however, they may cause adverse effects such as dependence and daytime drowsiness. Traditional Chinese medicine (TCM) offers a safer alternative. Zhi-zi-chi Decoction (ZZCD), composed of *Fructus gardeniae* and fermented soybean, has shown potential in promoting sleep, but its mechanism remains unclear.

**Methods:**

A PCPA-induced mice model of insomnia was used to evaluate the therapeutic effects of ZZCD. Behavioral tests, neurotransmitter assays, ELISA for HPA axis and inflammatory markers, 16S rDNA gut microbiota sequencing, and histological analyses of brain and colon tissues were performed.

**Results:**

ZZCD significantly improved sleep and reduced anxiety-like behavior. It restored GABA and 5-HT levels while lowering GLU and DA, normalized HPA axis activity, reduced inflammatory cytokines, and increased beneficial gut bacteria such as *Ligilactobacillus*. Histology confirmed neuroprotective and gut barrier-enhancing effects.

**Conclusion:**

ZZCD alleviates insomnia by modulating the gut-brain axis, rebalancing neurotransmitters, and restoring microbial homeostasis. These findings support its use as a safe and effective treatment for insomnia.

## 1 Introduction

Insomnia is a prevalent chronic sleep disorder encompassing difficulty initiating sleep, maintaining sleep, early awakening, or a combination of these types (Morin et al., 2021). It affects approximately 10–30% of adult population globally, significantly impairing quality of life and leading to adverse health consequences, such as cardiovascular disease, diabetes, depression, and cognitive dysfunction (Liu et al., 2016). The economic burden associated with insomnia is substantial, including medical costs and productivity losses, which making effective treatment has become a public health priority (Hafner et al., 2017). Current pharmacological treatments for insomnia, including benzodiazepines, non-benzodiazepine hypnotics, and melatonin receptor agonists, can provide short-term relief but often come with side effects such as dependency, tolerance, and residual daytime sleepiness (Riemann et al., 2017). Although cognitive behavioral therapy is a recognized first-line non-pharmacological treatment for insomnia (Sutton, 2021), its accessibility is limited by the availability of trained therapists and the high associated costs. Given these limitations, there is growing interest in alternative therapies for the treatment of insomnia, i.e., traditional Chinese medicine (TCM).

TCM offers a promising approach to treating insomnia by restoring balance and harmony within the body, thereby mitigating the side effects commonly associated with conventional pharmacological treatments (Feng et al., 2023). However, current TCM research on insomnia faces several limitations, including inconsistent clinical trial designs, lack of standardized protocols, and insufficient mechanistic evidence linking TCM formulations to specific neurobiological pathways (Ye et al., 2024). Additionally, while TCM is generally considered safer than synthetic drugs, the long-term safety profiles of certain herbal combinations remain understudied, and individual variability in treatment response poses challenges for widespread clinical application (Mssusa et al., 2023). Under this context, the distinctive advantages of the “medicinal food homology” concept become prominent. By integrating the nutritional and therapeutic properties of certain plants and animals, it provides dual-purpose interventions that align with TCM’s holistic philosophy (Ng et al., 2023). This approach not only supplies essential nutrients but also supports disease prevention and treatment, minimizing adverse effects typically associated with pharmaceutical drugs (Song and Jiang, 2017). In terms of safety, the side effects of consuming food are generally mild, whereas the use of medicines or drugs carries a higher risk of adverse effects. Zhi-zi-chi decoction (ZZCD), contains *Fructus gardeniae* (FG) and fermented soybean (FS), is a prime example of a TCM formulation that embodies the concept of medicinal food homology. FG comes from the dried mature fruit of *Rubiaceae* plant, recorded in China’s earliest TCM text, i.e., Shennong Bencao Jing (Zang et al., 2022). Normally, FG is used both as a seasoning food and a medicinal herb, known for its anti-inflammatory and sedative properties (Zhang et al., 2020). Similarly, FS has been well-documented for its health benefits, with soybeans used in TCM to alleviate restlessness and insomnia (Messina and Gleason, 2016). Therefore, this study primarily focuses on employing ZZCD to treat insomnia and investigates its therapeutic effects and the underlying mechanisms from the standpoint of the gut-brain axis.

Insomnia arises not only from central nervous system abnormalities but also from imbalances in the gut-brain axis. The gut-brain axis serves as a bidirectional signaling network linking gut microbiota with brain function (Kasarello et al., 2023). The gut microbiota encompasses the diverse microbial populations inhabiting the human intestine, which include bacteria, fungi, viruses, and protozoa. Dysbiosis within the gut microbiota can activate this particular axis, consequently modulating neural, endocrine, and immune response pathways (Adak and Khan, 2019), thereby affecting brain function and improving mood, cognitive performance, and sleep quality (Hu et al., 2016). On one hand, Metabolites produced by gut microbiota, including short-chain fatty acids (SCFAs), have been shown to inhibit excessive activation of the hypothalamic–pituitary–adrenal (HPA) axis (Dalile et al., 2020), resulting in decreased secretion of adrenocorticotropic hormone (ACTH) and cortisol (CORT) (Tofani et al., 2025). This subsequently interacts with GABAergic neurons, leading to an imbalance between glutamate (GLU) and *γ*-aminobutyric acid (GABA), lowering neuronal excitability and alleviating insomnia (Crowley and Girdler, 2014; Lo Martire et al., 2020). However, gut microbiota dysbiosis can disrupt the intestinal barrier, causing “leaky gut” (Dmytriv et al., 2024), which allows pro-inflammatory factors such as tumor necrosis factor alpha (TNF-*α*) and interleukin-1 (IL-1) to enter systemic circulation, triggering a systemic inflammatory response that contributes to insomnia (Li et al., 2023). On the other hand, the gut microbiota possesses metabolic capabilities, enabling it to break down substances like tyrosine into dopamine precursors (Wang et al., 2021). By modulating tyrosine hydroxylase activity, it influences central dopamine (DA) levels, which play a critical role in regulating motivation and reward systems (Cai et al., 2024), thereby exerting control over emotional states. This study is grounded in the mechanisms described above to investigate how ZZCD alleviates insomnia through these pathways (Murillo-Rodríguez et al., 2017; Legchenko et al., 2024).

The most commonly used method for modeling insomnia is intraperitoneal injection of DL-4-Chlorophenylalanine (PCPA). PCPA is a tryptophan hydroxylase inhibitor that suppresses the synthesis of 5-hydroxytryptamine (5-HT), which exerts a crucial influence on the induction and perpetuation of slow—wave sleep. This modeling method is stable and causes minimal harm to animals. Consequently, this method was employed to create an insomnia model. Subsequently, the efficacy and underlying mechanism of ZZCD in the treatment of insomnia were assessed by examining the diversity of gut microbiota, the levels of monoamine neurotransmitters, and the concentrations of hormones related to the HPA axis.

## 2 Materials and methods

### 2.1 Drugs and its preparation

ZZCD was obtained from Zhang Zhongjing Pharmacy in Henan, China. Diazepam (DZP) (H37023039) was bought from Shandong Xinyi Pharmaceutical Co. Ltd. PCPA was sourced from Shanghai Macklin Biochemical Co., Ltd. (Shanghai, China). Sodium pentobarbital (2018042001) was acquired from Huaxia Chemical Reagent Co., Ltd. (Sichuan, China). Enzyme-linked immunosorbent assay (ELISA) kits for various biomarkers including 5-HT, NE, DA, corticotropin-releasing hormone (CRH), ACTH, CORT, IL-1, and TNF-*α* were all purchased from Shanghai enzyme-linked biotechnology Co., Ltd. (Shanghai, China). For ZZCD preparation, 9 g FG and 8 g FS were soaked in 1000 mL distilled water for 1 h and then boiled for 1 h. After collecting the decoction, 800 mL of water was added and also boiled for 1 h for the second decoction. The two decoctions were combined, frozen, and then concentrated using a rotary evaporator at 65°C to prepare the ZZCD extract. Based on the conversion ratio between humans and mice, the concentrated volume of a single dose of ZZCD was calculated to be 41.8 mL. The suspension of DZP (1 mg/kg) and PCPA powder (60 mg/mL) was obtained by dissolving them in distilled water.

### 2.2 Establishment of animal model and administration of ZZCD

Male ICR mice (25–35 g) were purchased from Beijing HFK bioscience Co., Ltd. (China, Beijing, SYXK (Jing) 2024-0015) and the animals were kept in a controlled environment for 7 d. The room temperature stayed at 25 ± 2°C, the humidity at 50 ± 5%, and there was a 12 h light—dark cycle, with 12 h of light and 12 h of darkness daily. Approval for the animal experiments was bestowed by the Animal and Ethics Review Committee of Henan University of Chinese Medicine (approval number: IACUC-202410039). All male ICR mice were randomly allocated into four groups (*n* = 6 per group) using a computer-generated randomization sequence: control group, model group, ZZCD group, and DZP group (3 mg/kg of DZP). Except for control group, which was injected with 0.3 mL of saline, all other groups received a daily intraperitoneal injection of a PCPA suspension (300 mg/kg) once between 8:00 and 9:00 for 2 consecutive days to establish a insomnia model (Zhu et al., 2024). On the 9th day, mice injected with PCPA were observed to exhibit abnormal mental status, with rough fur and increased irritability. Between control group and model group, verification through a pentobarbital sodium sleep test found statistical differences in sleep latency and sleep duration. Subsequently, the corresponding groups were gavaged with 0.3 mL of respective samples once daily for 7 consecutive days. On the 16th day, the open field test and elevated plus maze test were conducted. On the 17th day, the pentobarbital sodium sleep test was performed and followed collecting the mice feces. On the 18th day, blood was collected from the orbital cavity, and the mice were euthanized by cervical dislocation after anesthesia with deep chloral hydrate. After euthanasia, the medulla oblongata was immediately severed at the foramen magnum, and mice’s skull was removed to fully expose the brain tissue. Half of brain was fixed for hematoxylin and eosin (H&E) and Nissl staining analysis, while the other half was collected on ice, separated, and stored in a refrigerator at −80°C.

### 2.3 Behavioral testing

3 d before the end of drug administration, mice underwent the open field test as follows: after 30 min of drug administration, the mice were first placed for 5 min to adapt to the environment. Afterward, mice were placed inside a 50 cm × 50 cm open field to collect behavioral parameters for 5 min. The arena was divided into a central zone (25 cm × 25 cm square in the middle) and a peripheral zone (the remaining area). Alcohol was used to eliminate the scent of previous mice before proceeding with the next experiment. The main parameters collected were the total distance moved (in cm) and the total activity time (in S). Similarly, 3 d before the end of drug administration, the mice underwent the elevated plus maze test. The elevated plus maze test is elevated 40 cm above the ground. The maze consists of two open arms and two closed arms (each arm = 30 cm, central area = 6 cm × 6 cm). The central zone in the EPM test serves both as the starting point of the experiment and an important reference area for behavioral observation. The time spent by mice in the central zone and the frequency of entries into each arm reflect their exploratory behavior and anxiety level. During the test, each mice was placed in the central zone facing an open arm and allowed to explore freely for 5 min. The number of entries and the time spent (in S) by mice in the open arms were recorded and analyzed.

### 2.4 Pentobarbital sodium sleep test

1 d before the end of drug administration, a pentobarbital sodium sleep experiment was conducted. 1 h after gavage, mice were intraperitoneally injected with pentobarbital sodium (48 mg/kg) and then placed supine on a cushion. Sleep latency (in S) and sleep duration (in min) were recorded. Sleep was defined as the loss of righting reflex, and awakening as its recovery. If uncertain, mice were placed back on their backs; if they flipped over within 1 min, the first flip time was recorded as the recovery time; otherwise, the second flip time was used.

### 2.5 Serum biochemical analysis and relevant indicators detection of hypothalamus

After 7 d of drug administration, all ICR mice were fasted for 12 h. Blood samples were collected via orbital bleeding and centrifuged using a high-speed centrifuge at 4°C (1,000 *g*, 20 min) to obtain serum. Using the serum samples, ELISA kits were used to detect the levels of CRH, ACTH, CORT, IL-1, and TNF-*α*, respectively. After euthanasia, hippocampal tissues of mice were promptly dissected and stored at −80°C. Subsequently, hippocampal tissues were thoroughly homogenized using a tissue homogenizer with phosphate-buffered saline at −4°C. After that, homogenates were centrifuged at 5000 *g* for 5 min, and the supernatants were collected. Following the instructions provided, ELISA kits were utilized to measure the levels of GABA and GLU. After the mice are euthanized, hypothalamic tissue is quickly isolated and stored at −80°C. Consequently, hypothalamic tissue was homogenized using a tissue grinder at a ratio of 1:9 with PBS at −4°C. After 5,000 g × 10 min centrifugation, supernatant is collected and the levels of monoamine neurotransmitters 5-HT, DA, and NE are measured using ELISA kits. All ELISA measurements were performed in duplicate, with an intra-assay coefficient of variation (CV) < 10%, ensuring the reliability of the results.

### 2.6 Gut microbiota analysis

Using 16S rDNA high-throughput sequencing, gut microbiota analysis of animal is conducted as follows: mice feces were subjected to total DNA extraction for microbial communities using the FastPure stool DNA isolation kit (MJYH, Shanghai, China). PCR amplification of full-length 16S rDNA gene was performed using primers 27F (5’-AGRGTTYGATYMTGGCTCAG-3′) and 1492R (5’-RGYTACCTTGTTACGACTT-3′) with barcodes, following an initial denaturation at 95°C for 3 min, 27 cycles of denaturation at 95°C for 30 S, annealing at 60°C for 30 S, and extension at 72°C for 30 S, followed by a final extension at 72°C for 10 min, and storage at 4°C (Weisburg et al., 1991). PCR products were detected by 2% agarose gel electrophoresis and then purified using AMPure PB beads (Pacific Biosciences, CA, United States). After that, PacBio raw reads were processed using the SMRTLink analysis software (version 11.0) to obtain high-quality Hifi reads with a minimum of three full passes and 99% sequence accuracy. Data from individual samples were distinguished based on barcode sequences, filtered by length, and sequences ranging from 1,000–1800 bp (for bacteria) were retained. Usearch 11 was used to cluster sequences into operational taxonomic units (OTUs) at a 97% similarity threshold and to remove chimeras (Edgar, 2013). To minimize the impact of sequencing depth on subsequent *α*- and *β*-diversity analyses, the sequence count for all samples was rarefied to 6,000. RDP classifier was used to annotate the OTU taxonomic classification by aligning against NT_16s (v20230830) with a confidence threshold of 70%, and the community composition of each sample was tallied at different taxonomic levels (Wang et al., 2007). Finally, PICRUSt2 software (version 2.2.0) was employed for 16S functional prediction analysis (Douglas et al., 2020).

### 2.7 Histological evaluation

Using H&E staining and Nissl staining, pathological examination of hypothalamic tissue was conducted. Brain tissues fixed in paraformaldehyde were retrieved and rinsed with PBS, and subsequently dehydrated with ethanol in a graded series. After embedding with wax, tissues were cut to continuous sections with a thickness of 5 μm and subjected to staining, dehydration, clearing, and mounting. This was achieved by treating the sections with increasing concentrations of ethanol solutions and xylene, ultimately leading to their mounting. The sections were stained separately with eosin and toluidine blue to observe the pathological changes in hippocampus and Nissl bodies in neuronal cells.

### 2.8 Statistical analysis

The statistical analysis was carried out utilizing SPSS 21.0 software, and the results were presented in the form of mean ±SD. The t-test and one-way ANOVA were employed to examine group differences (*p* value <0.05 was considered statistically significant). Graphs were generated using GraphPad Prism 9.0.

## 3 Results

### 3.1 Anxiety-relieving effect and sleep-alleviating effect of ZZCD on insomnia mice

**Figure 1:**
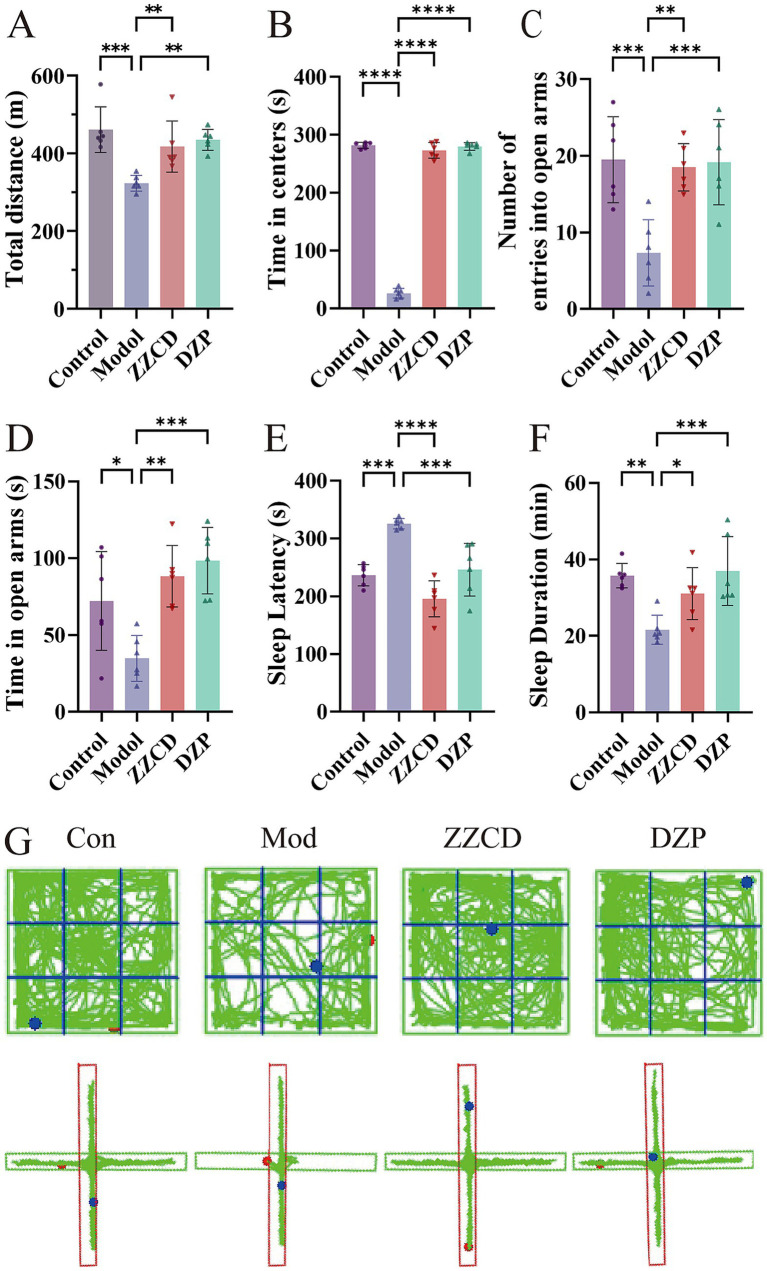
Effects of ZZCD on behavior and sleep in insomnia mice. **(A)** Total travel distance of mice. **(B)** Time spent in central area in mice. **(C)** Number of entries into the open arms in mice. **(D)** Time spent in open arms in mice. **(E)** Sleep latency in pentobarbital-induced sleep test. **(F)** Sleep duration in pentobarbital-induced sleep test. **(G)** Behavioral trajectory maps of mice in open field test and elevated plus maze test (^*^p < 0.05, ^**^p < 0.01, ^***^p < 0.001, ^****^p < 0.0001).

In the open field test, anxiety-related behavior was assessed by measuring the total distance moved and the time spent in the central zone. Mice in the model group exhibited significant decreases in both parameters relative to the control group (Figures 1A,B, *p < 0.001*). Mice receiving ZZCD treatment demonstrated a markedly higher overall distance covered and a considerably longer time period in the central region in comparison to the model group. Subsequently, elevated plus maze test was conducted on mice using the number of entries and the time spent in open arms as assessment indicators. Mice in the model group exhibited a significant reduction in both the frequency of open arm entries and the duration spent in open arms compared to the control group (Figures 1C,D, *p < 0.05*). Administration of ZZCD significantly enhanced open arm exploration, as evidenced by increased entry frequency and duration. The experimental results indicate that ZZCD can alleviate tension and anxiety-like behaviors in insomnia mice. Compared to control group, observations on sleep latency revealed a significant increase in model group (Figures 1E,F, *p < 0.001*). Both the ZZCD group and the DZP group displayed a notable decrease in sleep latency in comparison to the model group (*p < 0.001*). Sleep duration was significantly reduced in the model group relative to the control group (*p < 0.01*). Meanwhile, both ZZCD group and DZP group showed an increase in sleep duration (*p < 0.05*). ICR mice exhibited distinct locomotor trajectories across behavioral tests (Figure 1G).

### 3.2 Impact of ZZCD on HPA axis

**Figure 2:**
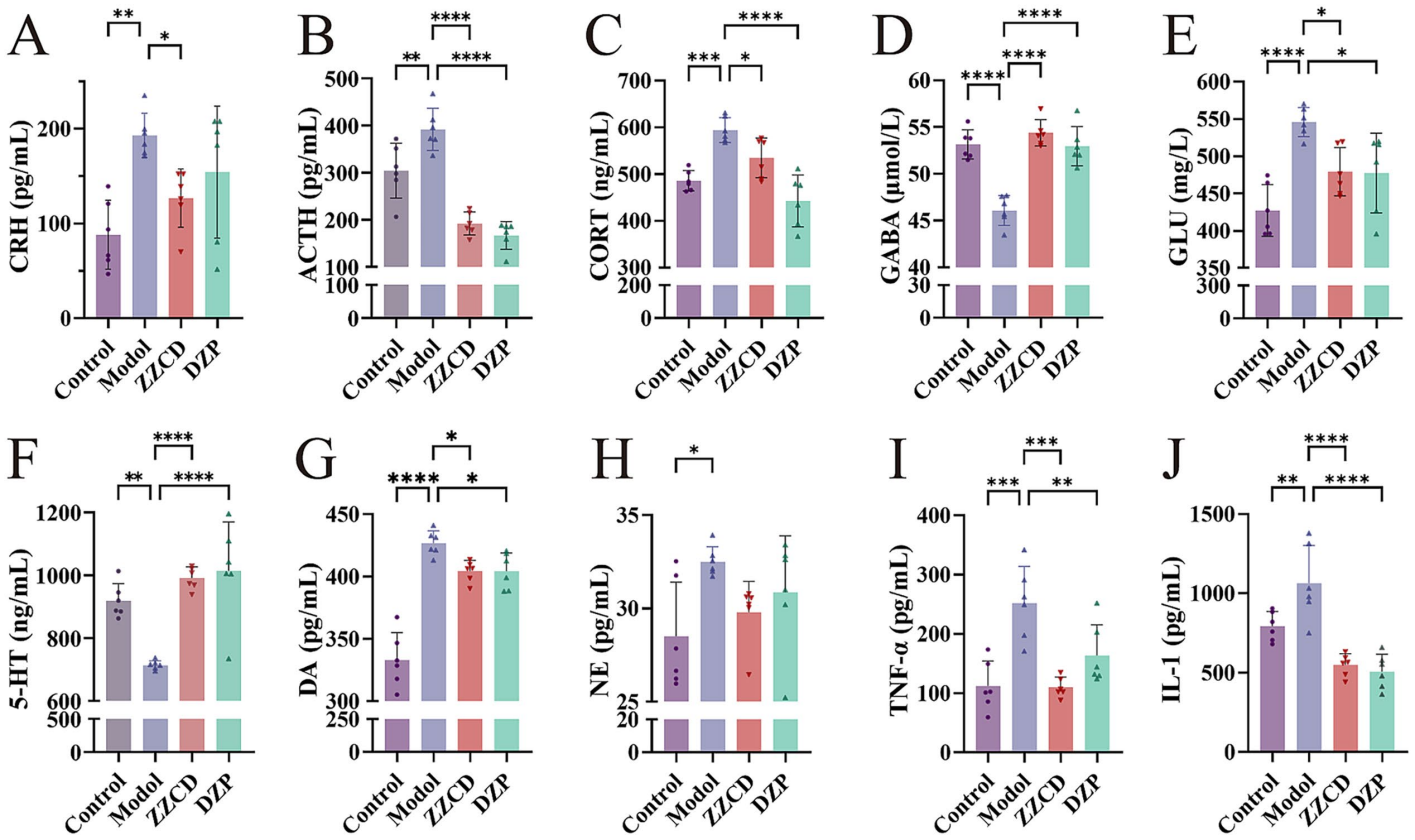
Effects of ZZCD on HPA axis, monoamine neurotransmitters, GABA system, and inflammatory factors in insomnia mice. **(A–C)** Represents the levels of CRH, ACTH, and CORT in mice serum, respectively. **(D–F)** Represents the levels of 5-HT, NE and DA in mice hypothalamus, respectively. **(G,H)** Represents the levels of GABA and GLU in the mice hippocampus, separately. **(I,J)** Represents the levels of TNF-*α* and IL-1 in mice serum, separately (^*^p < 0.05, ^**^p < 0.01, ^***^p < 0.001, ^****^p < 0.0001).

The levels of CRH, ACTH, and CORT in the serum were assessed using commercially available ELISA kits. The model group showed elevated serum levels of CRH, ACTH, and CORT relative to the control group (Figures 2A–C, *p < 0.01*). Administration of ZZCD led to a decrease in serum concentrations of CRH, ACTH, and CORT relative to the model group (*p < 0.05*). Simultaneously, DZP administration significantly lowered serum levels of ACTH and CORT (*p < 0.0001*). These findings imply that ZZCD might have an impact on the sleep condition of insomniac mice through regulating the HPA axis.

### 3.3 Impact of ZZCD on relevant indicators of hypothalamus

To determine the secretion levels of GABA and GLU, we measured their contents in hippocampus using ELISA kits. Relative to the control group, the model group showed notably lower levels of GABA in the hippocampus (Figures 2D,E, *p* < 0.0001), accompanied by an increase in GLU content (*p* < 0.0001). In view of this, ZZCD improves sleep quality in mice with insomnia, potentially by adjusting the secretion of GABA and GLU. By assessing the amounts of 5-HT, DA, and NE in the hypothalamus, this research intended to delve into the influence of ZZCD on neurotransmitters in insomniac mice. The model group showed elevated DA and NE levels (Figures 2G,H, *p* < 0.05), along with a reduction in 5-HT (Figure 2F, *p* < 0.01), relative to the control group. In comparison with the model group, both ZZCD and DZP markedly enhanced 5-HT content (*p* < 0.0001) and reduced DA levels (*p* < 0.05). These results imply a potential mechanism whereby ZZCD improves sleep quality through serotonergic activation and dopaminergic inhibition in a murine model of insomnia.

### 3.4 Impact of ZZCD on inflammatory cytokines

To examine the inflammatory profile associated with insomnia, we evaluated serum TNF-*α* and IL-1 concentrations. As shown in Figures 2I,J, TNF-α and IL-1 levels were significantly higher in the model group than in the control group (*p* < 0.01). Treatment with ZZCD resulted in a significant decrease in TNF-α and IL-1 levels (*p* < 0.001). Thus, we can conclude that ZZCD improves sleep in mice by modulating the secretion of TNF-α and IL-1.

### 3.5 Analysis of gut microbiota

#### 3.5.1 Analysis of α- and *β*-diversity

**Figure 3:**
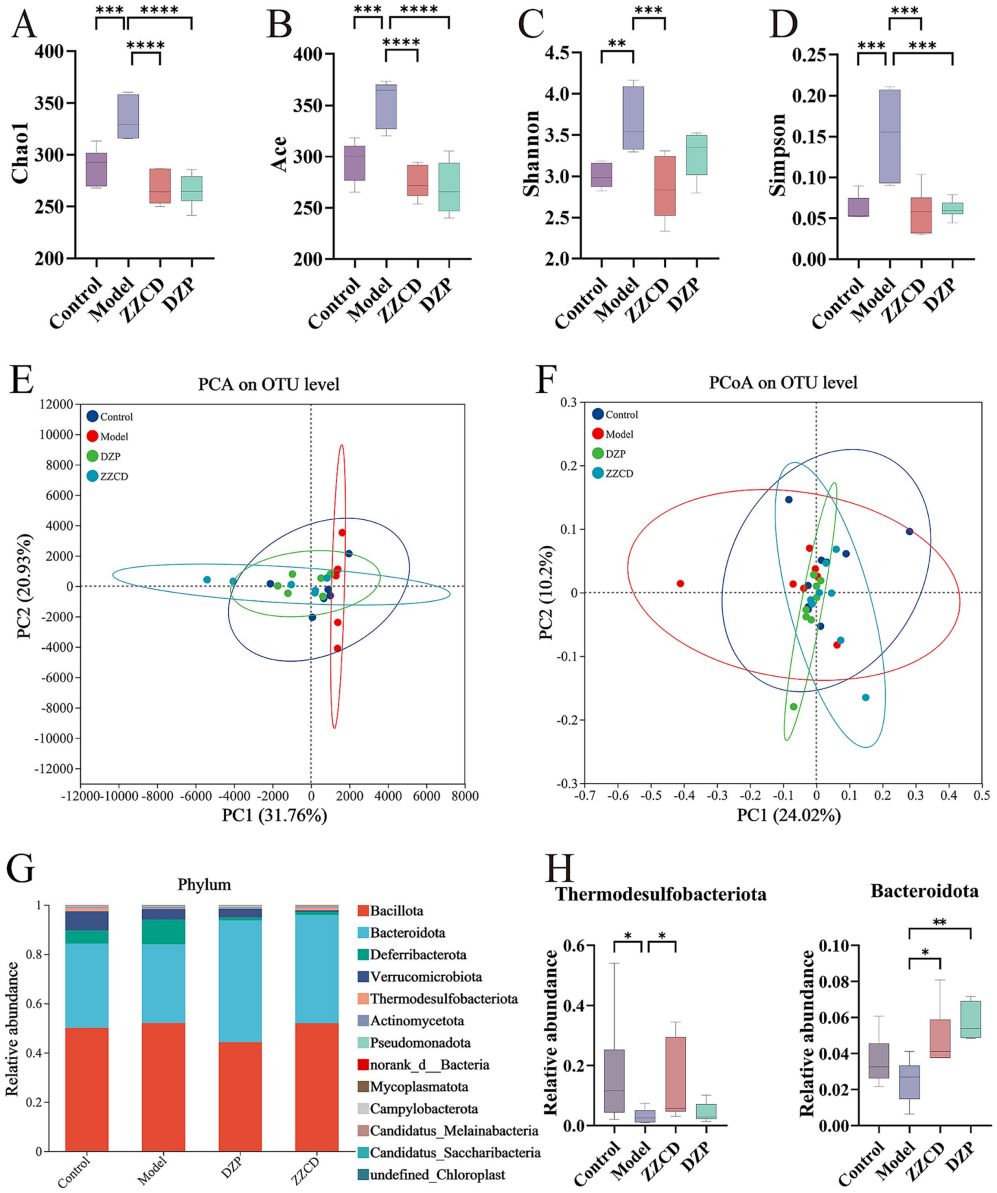
Effects of ZZCD on abundance, composition, and phylum-level distribution of gut microbiota in insomnia mice. **(A–D)** Represents the index of Chao1, ACE, Shannon, and Simpson, respectively. **(E,F)** Represents the PCA plot and PCoA plot separately. **(G)** Bar graph illustrating the relative abundance of species at the phylum taxonomic level. **(H)** Species significantly influenced by ZZCD at the phylum taxonomic level in insomniac mice (^*^p < 0.05, ^**^p < 0.01, ^***^p < 0.001, ^****^p < 0.0001).

The Chao1 and ACE indices, indicators of microbial richness, showed a direct correlation between index magnitude and species abundance. Shannon and Simpson indices quantify the diversity of the gut microbiome by incorporating species richness and evenness; higher scores reflect more complex communities (Williams et al., 2024). The model group showed significantly higher values in Chao1, ACE, Shannon, and Simpson diversity indices relative to the control group (Figures 3A–D, *p* < 0.01). After administration of different doses of ZZCD, these indices decreased to levels consistent with the control group. This suggests that ZZCD treatment restored the gut microbiota abundance and diversity in insomnia mice to levels comparable to those of healthy controls. For the β-diversity analysis, the model group exhibited an upward deviation compared to control and ZZCD groups (Figure 3E). Nonetheless, Statistical analysis revealed no significant differences across the control, model, and ZZCD groups (Figure 3F). These results demonstrated noticeable shifts in microbial community structure between untreated insomnia mice and those receiving the intervention.

#### 3.5.2 Analysis of gut microbiota composition

Figures 3G,H present microbial taxonomic distributions at both the phylum and genus levels, emphasizing the top 30 taxa and highlighting statistically significant variations. As shown in Figure 3G, *Bacillota* and *Bacteroidota* emerged as the predominant phyla. Compared to control group, model group showed increased relative abundances of *Bacteroidota*, *Verrucomicrobiota*, *Thermodesulfobacteriota*, and *Pseudomonadota*, while the relative abundances of *Verrucomicrobiota*, *Thermodesulfobacteriota*, *norank_d__Bacteria*, and *Campylobacterota* decreased. After ZZCD treatment, except for *Thermodesulfobacteriota* and *Pseudomonadota*, the relative abundances of all other phyla showed varying degrees of reversal. Among them, *Bacteroidota* and *Thermodesulfobacteriota* exhibited significant differences in ZZCD group (*p* < 0.05).

**Figure 4:**
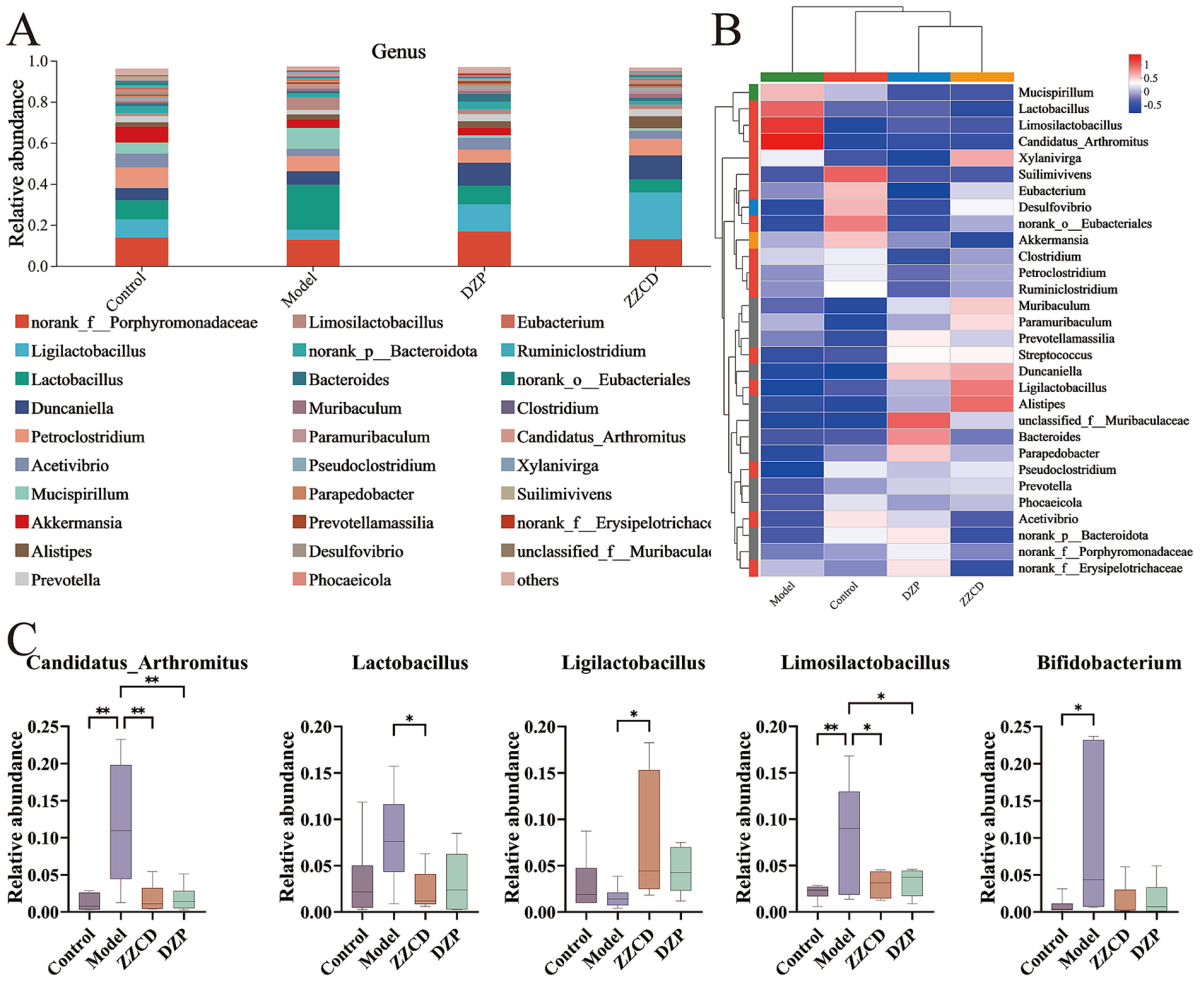
Effects of ZZCD on genus-level gut microbiota in insomnia mice. **(A)** The relative abundance of species at the genus taxonomic level. **(B)** Intergroup cluster analysis of the relative abundances of taxa at genus level. **(C)** Species significantly influenced by ZZCD at the genus taxonomic level in insomniac mice (^*^p < 0.05, ^**^p < 0.01).

In Figure 4A, *norank_f__Porphyromonadaceae* and *Ligilactobacillus* emerged as the dominant genera. To reflect the similarities and differences among different taxa as a whole, a heatmap was used for analysis. As shown in Figure 4B, the control group, ZZCD group, and DZP group exhibited a clustering pattern. Compared to the model group, both the ZZCD and DZP treated groups exhibited similar color distribution patterns, suggesting their efficacy in restoring gut microbiota homeostasis in insomnia mice. Compared to control group, model group showed significant decreases in *norank_f__Porphyromonadaceae*, *Ligilactobacillus*, *Acetivibrio*, *Akkermansia*, *norank_p__Bacteroidota*, *Parapedobacter*, and *Desulfovibrio*, while *Lactobacillus*, *Limosilactobacillus*, *Paramuribaculum*, *Candidatus_Arthromitus*, and *Bifidobacterium* increased significantly. Except for *Paramuribaculum* and *Desulfovibrio*, the relative abundances of all other taxa showed varying degrees of changes. ZZCD treatment significantly restored the relative abundances of gut microbiota including *Lactobacillus*, *Ligilactobacillus*, *Limosilactobacillus*, *Bifidobacterium*, and *Candidatus_Arthromitus* to levels comparable with the control group (Figures 4C, *p < 0.05*).

#### 3.5.3 Analysis of gut microbiota biomarkers

**Figure 5:**
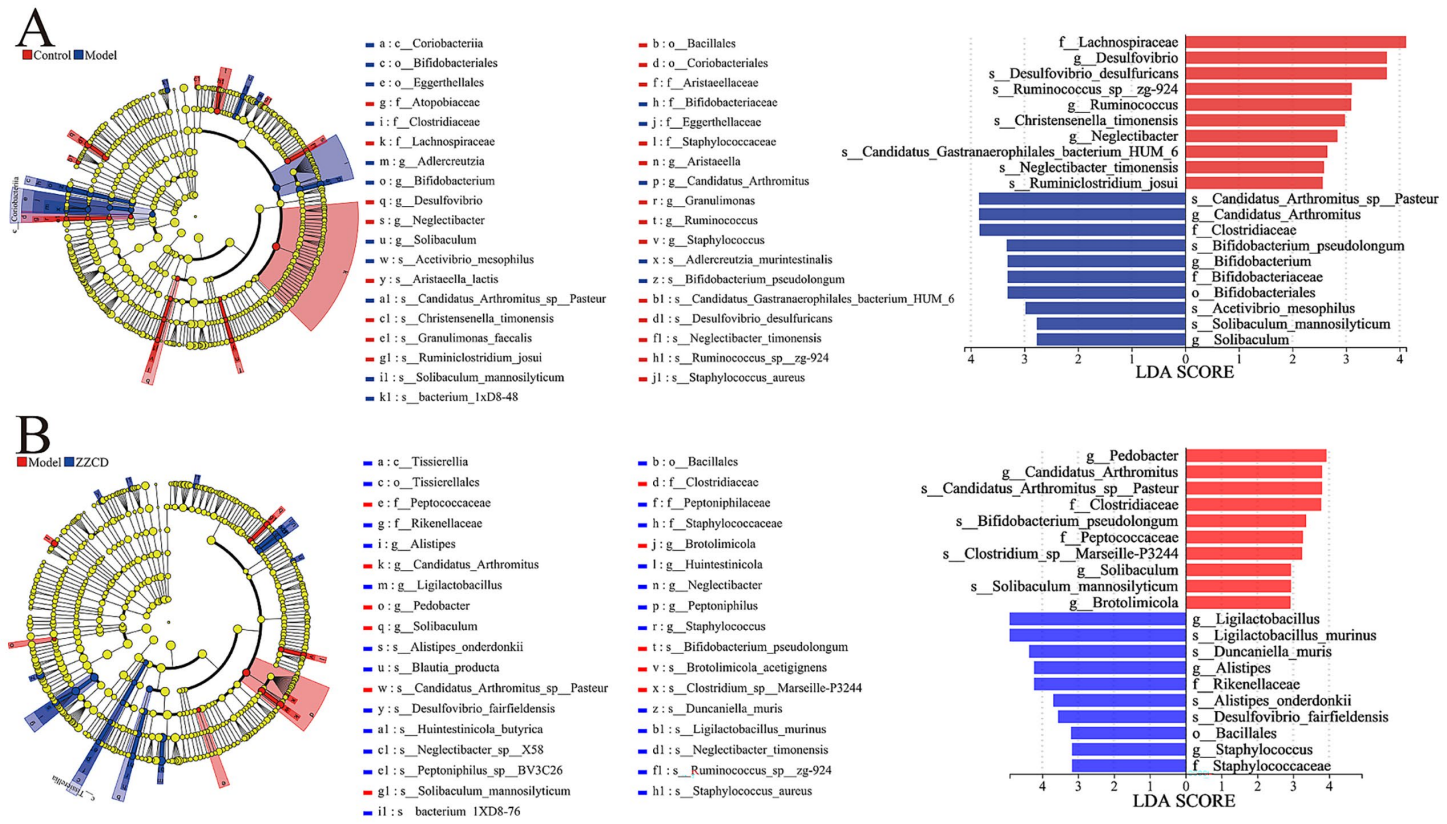
Comparison of gut microbiota between different groups using LDA and LEfSe. **(A)** LDA score and cladogram of control group vs. model group generated by LEfSe analysis. The cladogram’s circles radiating from the inside out represent the classification level from phylum to species. **(B)** LDA score and cladogram of model group vs. ZZCD group generated by LEfSe analysis. The length of bar chart represents the impact of different species.

LEfSe analysis of the gut microbiota revealed significant differences between the normal and model groups, as well as between the model and ZZCD groups. Across these groups, only biomarker taxa with statistical significance (LDA threshold>3.0*, p < 0.05*) were determined. In Figure 5A, LEfSe analysis revealed a total of 12 biomarker taxa when betweeen control group and model group, which included *Lachnospiraceae*, *Desulfovibrio*, *Desulfovibrio*_*desulfuricans*, *Ruminococcus_sp__zg-924*, and *Ruminococcus* in control group, meanwhile, *Candidatus_Arthromitus_sp__Pasteur*, *Candidatus_Arthromitus*, *Clostridiaceae*, *Bifidobacterium_pseudolongum*, *Bifidobacterium*, *Bifidobacteriaceae*, and *Bifidobacteriales* in model group. In Figure 5B, between the model group and the ZZCD group, LEfSe analysis identified a total of 17 biomarker taxa, which included *Pedobacter, Candidatus_Arthromitus_sp__Pasteur*, *Candidatus_Arthromitus*, *Clostridiaceae*, *Bifidobacterium_pseudolongum*, *Peptococcaceae*, and *Clostridium_sp__Marseille-P3244* in model group, while ZZCD group contained *Ligilactobacillus*, *Ligilactobacillus_murinus*, *Duncaniella_muris*, *Rikenellaceae*, *Alistipes*, *Alistipes_onderdonkii*, *Desulfovibrio_fairfieldensis*, *Bacillales*, *Staphylococcus_aureus*, and *Staphylococcaceae*. The results indicated that ZZCD administration led to an elevated relative abundance of *Ligilactobacillus* in the intestinal microbiota of insomnia mice, a genus linked to insomnia-related pathology.

#### 3.5.4 Correlation analysis

**Figure 6:**
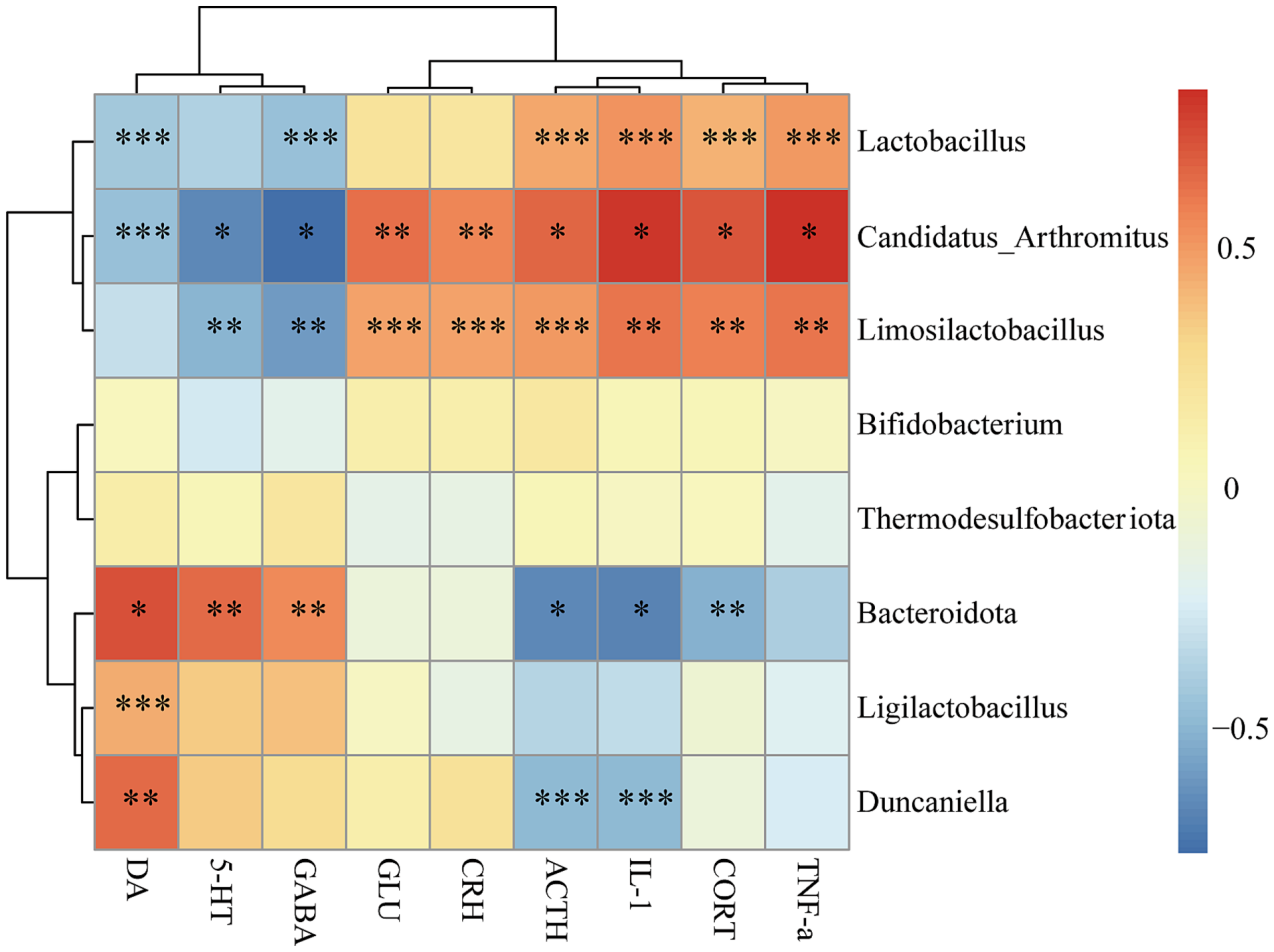
Correlation analysis between gut microbiota and biochemical markers. Correlation analysis between the biochemical markers (DA, 5-HT, GABA, GLU, CRH, ACTH, CORT, TNF-α, and IL-1) and gut microbiota at the phylum and genus taxonomic level was performed using Spearman correlation analysis. Red color represents positive correlations, blue color represents negative correlations. The color intensity corresponds to the magnitude of correlation coefficient, with darker shades indicating stronger correlations and lighter shades representing weaker correlations (^*^p < 0.05, ^**^p < 0.01, ^***^p < 0.001).

Functional correlations between biochemical markers and gut microbiota were conducted with Pearson correlation analysis, and the results were visually presented in form of a heatmap. As shown in Figure 6, *Lactobacillus*, *Candidatus_Arthromitus*, and *Limosilactobacillus* exhibited strong positive correlations with TNF-*α*, IL-1, ACTH, and CORT, while they were negatively correlated with GABA, 5-HT, and DA. Among them, *Candidatus_Arthromitus* and *Limosilactobacillus* showed highly positive correlations with CRH and GLU. *Bacteroidota* and *Duncaniella* were negatively correlated with ACTH and IL-1 but positively correlated with DA. Notably, *Bacteroidota* exhibited a significant positive association with both GABA and 5-HT, whereas its relationship with CORT was markedly negative. These findings indicate significant associations between biochemical markers and gut microbiota.

### 3.6 Pathological detection of hippocampus

**Figure 7:**
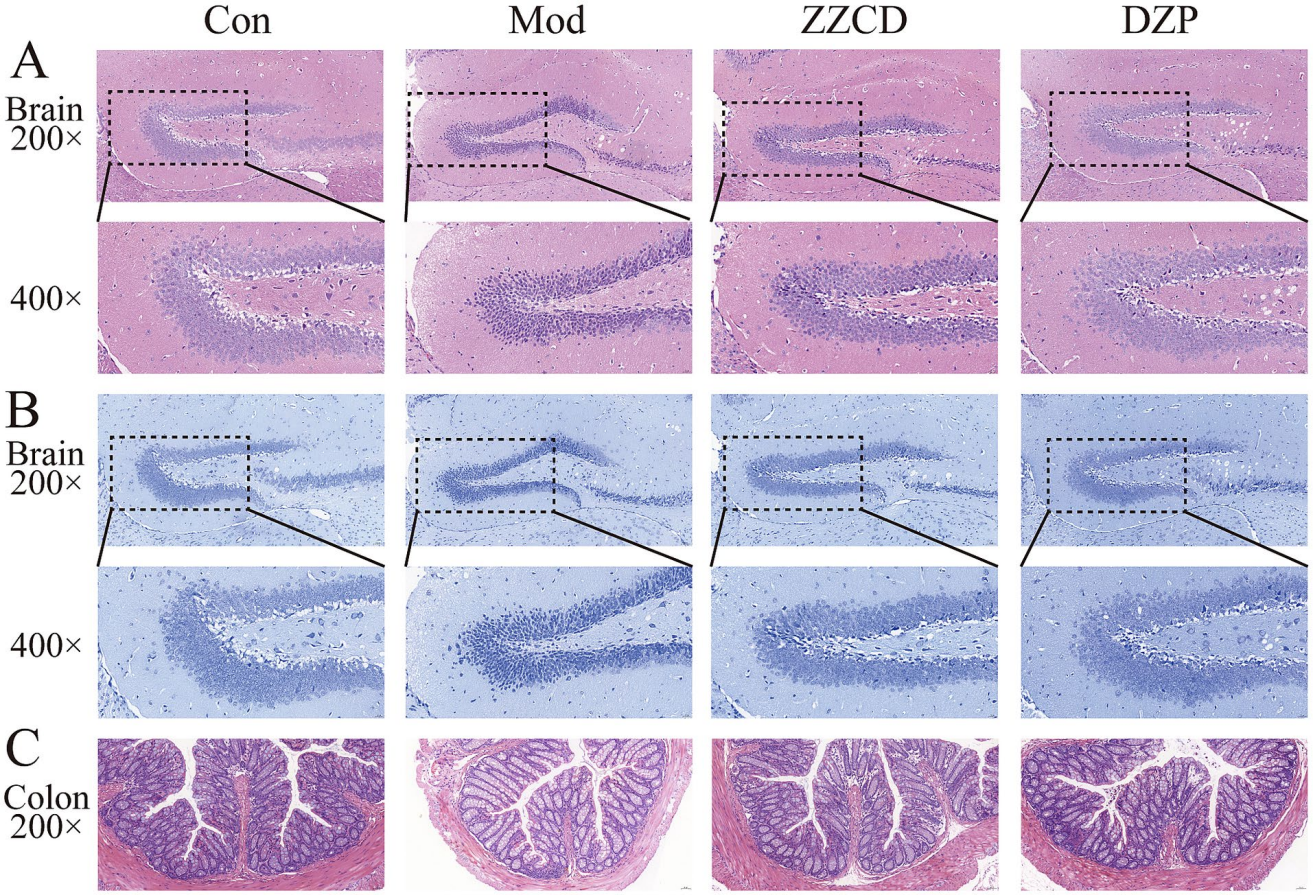
Effects of ZZCD on histopathology of brain and colon in insomnia mice. **(A)** HE staining of brain tissue (upper panel 200×, lower panel 400×); **(B)** Nissl staining of brain tissue (upper panel 200×, lower panel 400×); **(C)** HE staining of colon tissue (200×).

As shown in Figure 7, H&E staining and Nissl staining indicate that hippocampal neurons in control group mice are tightly and neatly arranged, with regular morphology and clearly visible Nissl bodies. In contrast, hippocampal neurons in model group mice exhibit disordered arrangement, reduced neuron number, and a large number of darkly stained neurons. Additionally, nuclear pycnosis, cytoplasmic dissolution, enlarged intercellular spaces, disordered arrangement, and decreased Nissl bodies are observed in model group. Compared with model group, both ZZCD group and DZP group show an increase in number of hippocampal neurons, with neatly arranged cells. Occasionally, darkly stained nuclei and relatively intact cell membranes are seen, indicating improvements in cell morphology and structure, along with an increase in Nissl bodies. These findings suggest that ZZCD has a certain repair effect on hippocampal neurons and Nissl bodies in insomnia mice induced by PCPA. Model group exhibited an increased number of inflammatory cells in colon, damaged villi, slight exfoliation of mucosal epithelial cells, loose arrangement of intestinal glands in lamina propria, and a thinned basement membrane. Compared to model group, ZZCD treatment significantly reduced colonic inflammatory cell infiltration, restored villi to levels comparable with the normal group, and induced structural improvements including compactly arranged lamina propria intestinal glands and a markedly thickened basement membrane.

## 4 Discussion

This study demonstrated that the therapeutic effects of ZZCD in treating insomnia are largely mediated through modulation of the gut microbiota. Specifically, ZZCD effectively ameliorated PCPA-induced insomnia in mice by modulating the gut-brain axis. Our findings support the hypothesis that ZZCD exerts its hypnotic and anxiolytic effects through the restoration of gut microbial balance, normalization of neurotransmitter systems, and attenuation of neuroendocrine dysregulation via the HPA axis. The previous studies showed that *Ligilactobacillus* could act as a promising biomarker in the context of insomnia therapy. The positive correlation between *Ligilactobacillus* abundance and levels of 5-HT and GABA supports the hypothesis that gut metabolites regulate central neurotransmission. *Ligilactobacillus* is known to play a critical role in maintaining intestinal barrier integrity, producing SCFAs, and modulating host immune responses, thereby contributing to gut homeostasis and influencing neuropsychiatric health. This suggests that ZZCD improves insomnia indirectly by modulating *Ligilactobacillus* to enhance 5-HT and GABA levels and to strengthen intestinal barrier and anti-inflammatory defenses. Furthermore, the reduced *α*-diversity in insomnia model and its reversal by ZZCD indicate that microbial community stability is critical for sleep regulation, likely through maintaining intestinal barrier integrity to prevent systemic inflammation, thereby ameliorating insomnia (Mishra et al., 2023). Following ZZCD treatment, a significant elevation in *Ligilactobacillus* abundance was observed; this genus plays a key role in gut metabolism and immune function, and its reduction has been associated with neuropsychiatric conditions (Chuandong et al., 2024). Oral administration of ZZCD increased GABA and decreased GLU in the hypothalamus, indicating restoration of the excitatory-inhibitory balance-a key determinant of the sleep–wake cycle. Notably, while both DZP and ZZCD showed nearly identical effects on neurotransmitters, only ZZCD modulated hippocampal 5-HT, significantly increasing its levels. This highlights ZZCD’s unique ability to enhance serotonergic signaling, a pathway crucial for maintaining slow-wave sleep (Jones, 2020).

The reduction in systemic pro-inflammatory cytokines and improved colonic inflammatory infiltration suggest that ZZCD disrupts the inflammation-insomnia vicious cycle. Histological improvements in hippocampal neurons and Nissl bodies further imply neuroprotective effects, potentially mediated through GABAergic system regulation. These findings extend prior reports on the anti-inflammatory properties of FG (Liu et al., 2023), demonstrating its synergistic effects with fermented soybeans in this complex formulation.

While this study confirms ZZCD’s efficacy in male mice; however, given the sex differences in insomnia prevalence, future investigations should include female subjects to comprehensively evaluate potential sex-specific effects. Although 16S rDNA sequencing effectively characterizes alterations in microbial composition, its inherent limitations restrict functional interpretation of microbial metabolites. Therefore, integration of metagenomic and metabolomic analyses in future studies is warranted to elucidate specific microbial pathways and functional mechanisms underlying ZZCD’s effects. Despite these promising results, future research is needed to further elucidate the molecular mechanisms of ZZCD, particularly to identify the active components involved in gut microbiota modulation.

## 5 Conclusion

This study demonstrated that ZZCD can significantly improve the insomnia symptoms through regulating the gut-brain axis. Specifically, ZZCD modulates the gut microbiota structure by promoting the proliferation of beneficial bacteria, such as *Ligilactobacillus*, which in turn indirectly increases the levels of 5-HT and GABA while reducing GLU levels. By restoring the balance between excitatory and inhibitory neurotransmitters in brain, ZZCD improves sleep quality and alleviates insomnia symptoms. Furthermore, restoration of microbial diversity and improvement of gut health further enhance sleep quality.

## Data Availability

The original contributions presented in the study are publicly available. This data can be found here: https://www.ncbi.nlm.nih.gov/bioproject/PRJNA1299127/.
